# Editorial: Wheat biofortification to alleviate global malnutrition

**DOI:** 10.3389/fnut.2022.1001443

**Published:** 2022-09-16

**Authors:** Maria Itria Ibba, Om Prakash Gupta, Velu Govindan, Alexander Arthur Theodore Johnson, Henrik Brinch-Pedersen, Miroslav Nikolic, Victor Taleon

**Affiliations:** ^1^Wheat Chemistry and Quality Laboratory, International Center for the Improvement of Wheat and Maize (CIMMYT), Texcoco, Mexico; ^2^Division of Quality and Basic Sciences, ICAR-Indian Institute of Wheat and Barley Research, Karnal, India; ^3^Global Wheat Breeding, International Center for the Improvement of Wheat and Maize (CIMMYT), Texcoco, Mexico; ^4^School of Bioscience, University of Melbourne, Parkville, VIC, Australia; ^5^Department of Agroecology, Aarhus University, Aarhus, Denmark; ^6^Institute for Multidisciplinary Research, University of Belgrade, Belgrade, Serbia; ^7^International Food Policy Research Institute, Washington, DC, United States

**Keywords:** wheat biofortification, malnutrition, micronutrient, bioavailability, GWAS—genome-wide association study

According to the latest FAO report on the state of food security and nutrition in the world (1), more than 720 million people faced hunger, and around 3 billion people did not have access to a healthy diet. All these problematics, exacerbated by the current COVID-19 crisis, led to an increase in the number of people affected by the so-called hidden hunger, caused by an inadequate intake of essential micronutrients (MNs) such as iron (Fe), zinc (Zn), selenium (Se) and provitamin A. Biofortification, intended as the improvement of the nutritional quality of food crops through either conventional breeding, agronomic practices or modern biotechnologies, represents a sustainable, cost-effective and long-term approach to alleviate micronutrient-deficiency. Staple crops are typically the major target of most biofortification studies, given their central role in human diet. Wheat, specifically, contributes to around 20% of the total energy and protein intake and to around 30% of the Fe and Zn intake worldwide. However, the current level of MNs present in most wheat-derived food products is not enough to meet the minimum daily intake, especially in the poorest regions of the world. For these reasons, continuing to work on wheat biofortification is fundamental to ensure the production of nutritious and sustainable food and to contribute to the reduction of MNs deficiency.

This special issue of Frontiers in Nutrition presents some of the most recent discoveries on wheat biofortification with studies spanning from the development of genetic tools to speed up conventional breeding, genetic engineering and novel agronomic methods to increase the MNs content in wheat grain. In this issue, Wang Y. et al. report on the identification of different quantitative trait loci (QTL) associated with variation in the grain Fe and Zn content using a bread wheat recombinant inbred line (RIL) population grown across nine different environments. Results of this study revealed the presence of seven different genomic regions associated with grain Zn content (explaining 2.2 to 25.1% variation), and four genomic regions associated with grain Fe accumulation (explaining 2.3 to 30.4% variation). Interestingly, three of the QTL identified in this study appeared to be associated with the accumulation of both Fe and Zn content. These QTL were therefore transformed into high-throughput Kompetitive Allele Specific PCR (KASP) markers that could be readily used to speed-up biofortification within a conventional breeding program. Similarly, Krishnappa et al. also investigated the genetic control of Fe and Zn accumulation in wheat grains using a RIL population grown in several environments. In this case, however, additional traits associated with grain quality (grain protein content and thousand kernel weight) were also included. Thanks to the high marker density and to the high D-genome coverage, several QTL associated with either Fe, Zn and protein content and thousand kernel weight could be identified. Among them, several were located on the D genome, with the chromosome 7D harboring several QTL associated with all the analyzed traits. Putative candidate genes responsible for the observed phenotypic variation were also identified, paving the road for more detailed future studies which would likely allow the characterization of the specific gene(s) responsible for the variation of these essential traits.

Identification of the genes (and relative enzymes) directly responsible for the accumulation, bioavailability or degradation of anti-nutrients, is undoubtly the final goal of most genetic studies as it could greatly facilitate genetic biofortification though either conventional breeding or transgenic approaches. For this reason, Yu and Tian reported the role of the Carotenoid Cleavage Dioxygenase 4 (CCD4) gene on the wheat grain carotenoid accumulation using a set of Targeting Induced Local Lesions in Genomes (TILLING) durum wheat lines. Results revealed that the CCD4 homeolog genes do not appear to have a significant impact on grain carotenoid content even if changes in the carotenoid composition could be identified in both wheat grains and leaves.

The idneitification of genomic regions associated with higher MN contents and development of genetic tools for the fast transfer of the high MN traits, are not the only approaches used to facilitate the development of MN rich wheat. Several studies have indeed shown that agronomic biofortification is an efficient and effective method to increase wheat grain micronutrient content in the short-term, especially if combined with genetic biofortification (Gupta et al.). Here, Gupta et al. have comprehensively reviwed the recent progress made in utilizing natural genetic diversity, genome-wide association mapping, genomic selection, and genome editing technologies to improve the MN content and their bioavailability in wheat. Yu et al. studied the potential of Zn foliar application on the wheat grown in the Quzhou County of China, a region where more than 90% of the population is engaged in agriculture and where ~39% of the children suffer from Zn deficiency. The result indicated that compared to control, wheat with Zn foliar application had 97.7 and 68.2% higher Zn content in wheat grain and flour, respectively withoput a significant change in wheat yield. However, according to the author's prediction, implementing this practice could significantly increase the daily Zn intake, reduce the disability-adjusted life years (DALYs) for both infants and children, and increase the overall economic income of this Chinese region. Similar results were reported by Hafeez et al. who investifgated the effect of soil Fe, Zn or Fe and Zn application, on the overall grain MN accumulation, grain quality and plant performance on Zn-efficient wheat variety (Zincol-16) and Zn-inefficient variety (Anaj-17). As expected, the plants grown on the soil treated with either of the three applications, exhibited significantly higher MN content and yield compared with the controls. Also, the Zn-efficient variety was able to accumulate higher Zn and Fe content, confirming that the combination of genetic and agronomic biofortification is an effective strategy to improve MN intake.

Even if agronomic biofortification has been widely proven to be an efficient biofortification approach, the dynamics of absorption and translocation of the applied MNs are complex and influenced by several factors including the chemical form of the MNs, application rate and its method of application. Ramkissoon et al. reported on the time-dependent changes in the absorption, transformation and distribution of Se applied to wheat leaves at two growth stages with or without the inclusion of urea. Results revealed that, independent of the treatment, grain Se content increased to a level adequate for biofortification even if the time of application and the presence of nitrogen (N) in the formulation significantly influenced the assimilation of Se. More studies focused on the optimization of the formulation of Se and other MN fertilizers, and on the best methods and timing of application will be fundamental to refine current agronomic biofortification practices and to optimize the micronutrient accumulation in grain.

When considering agronomic biofortification, the possible detrimental effect that this practice could have on the environment should also be considered. Microbial-assisted biofortification could be a solution to combine the short-term effects of the classic agronomic biofortification while reducing the negative impact that the increased fertilizer application could have on both soils and waters. Using an endophytic strain of *Bacillus altitudinis*, Sun et al. confirm the potential of this technique for wheat Fe biofortification. In this innovative study, the authors test two different inoculation methods and proved that, especially after spraying the microbe inoculum in the soil, the grain Fe accumulation significantly increased and that this *B. altitudinis* strain could efficiently colonize and translocate within wheat. Even if more studies will enable to understand the benefits and effectiveness of this method, microbial-assisted biofortification is an exciting emerging area of investigation that could significantly help create more nutrient-rich and sustainable grains.

Wheat biofortification however could not be enough if most of the micronutrients accumulated in the grains are lost during food processing. Before being consumed in fact, wheat grains are typically milled into refined flour and the bran, which is the part of the grain with the highest mineral content, is typically discarded as a by-product of the milling process. For this reason, Wang H. et al. investigated the effect of bran size and quantity on the quality of Chinese steamed bread, a staple food typically produced with refined flour. Results of this study revealed that addition of 5% wheat bran with medium particle size (D50 = 122.3 ± 7.1 μm) was able to significantly increase the Zn content present in the final product while maintaing an acceptable end-use quality. Nevertheless, independently from the utilization of refined or whole meal flour, regular adoption of biofortified wheat varieties is associated with an increased intake of zinc. As reported by Lowe et al. in fact, regular consumption of food products obtained from biofortified wheat, is associated with a 30% to 60% increased daily zinc intake. These results were obtained using an individually-randomized, double-blind, placebo-controlled cross over design and recruiting 50 households representative of a population with widespread zinc deficiency.

To conclude, development of nutrient-dense wheat has the potential to mitigate the micronutrient deficiency problems that affect a significant part of the world population, typically from developing or under-developed countries. Up to now, tremendous progresses have been made on wheat biofortification, but new knowledge and innovations must be generated to ensure a significant reduction in the incidence of hidden hunger. The studies presented in this issue well represent the latest discoveries and approaches proposed to increase the wheat micronutrient content, underlying the importance of addressing biofortification from different angles by combining both genetic and agronomic approaches.

## Author contributions

All authors listed have made a substantial, direct, and intellectual contribution to the work and approved it for publication.

## Conflict of interest

The authors declare that the research was conducted in the absence of any commercial or financial relationships that could be construed as a potential conflict of interest.

## Publisher's note

All claims expressed in this article are solely those of the authors and do not necessarily represent those of their affiliated organizations, or those of the publisher, the editors and the reviewers. Any product that may be evaluated in this article, or claim that may be made by its manufacturer, is not guaranteed or endorsed by the publisher.
